# Variations on Ogden’s model: close and distant relatives

**DOI:** 10.1098/rsta.2021.0322

**Published:** 2022-10-17

**Authors:** A. E. Ehret, A. Stracuzzi

**Affiliations:** ^1^ Empa, Swiss Federal Laboratories for Materials Science and Technology, Überlandstrasse 129, 8600 Dübendorf, Switzerland; ^2^ ETH Zurich, Institute for Mechanical Systems, Leonhardstrasse 21, 8092 Zürich, Switzerland

**Keywords:** Ogden’s model, hyperelastic materials, rubber elasticity, molecular statistical theory, phenomenological models

## Abstract

The power law in terms of stretch, the truncated series representation and the Valanis–Landel hypothesis are distinguished features of Ogden’s strain-energy density function. While they represent a set of special constitutive choices, they have also been shown recently to allow a particular molecular statistical interpretation of the model, where each of these ingredients can be associated with a step in the development of the strain-energy density of the polymer network from the statistical mechanics of long-chain molecules. The schematic of this perspective brings us into a position to vary these steps individually. By this means, Ogden’s theory can be embedded in a certain family of models within the large class of isotropic hyperelastic materials, whose members can be identified as close and distant ‘relatives’.

This article is part of the theme issue ‘The Ogden model of rubber mechanics: Fifty years of impact on nonlinear elasticity’.

## 1. Introduction

In 1972, R. W. Ogden proposed a new class of strain-energy density functions for incompressible and compressible rubber-like materials [1,2], the first of which has become probably one of the most used constitutive equations for highly deformable, isotropic hyperelastic materials to date. The proposed form differed from the common approaches at that time, and even nowadays its development in terms of the principal stretches stands out from the majority of phenomenological hyperelastic models, which are formulated in terms of the principal strain invariants. Clearly, the invariants can be expressed as quadratic functions of the principal stretches (see e.g. [3]), whereas the squared principal stretches are the roots of the characteristic polynomial in terms of the principal invariants [4,5]. Accordingly, any invariant-based strain-energy density function can generally be expressed in terms of the principal stretches and vice versa. However, the corresponding reformulation generally leads to more complicated mathematical expressions, which can become particularly cumbersome in the analysis of stress and stiffness. In fact, simplicity of mathematical analysis was an explicit argument for the development of Ogden’s incompressible model [1]

1.1 $$\Psi =\sum_{r=1}^{R}\frac{\mu_{r}}{\alpha_{r}}(\lambda_{1}^{\alpha_{r}}+\lambda_{2}^{\alpha_{r}}+\lambda_{3}^{\alpha_{r}}-3)=\sum_{i=1}^{3}\sum_{r=1}^{R}\frac{\mu_{r}}{\alpha_{r}}(\lambda_{i}^{\alpha_{r}}-1),\;\lambda_{1}\lambda_{2}\lambda_{3}=1,$$

with $\mu_{r}\alpha_{r}>0$ (no sum) for each r. Indeed, given that stress and strain share the same principal directions in isotropic hyperelastic materials, the form (1.1) leads to particularly lean expressions for the principal stresses, which facilitate comparison of the model response under ideal homogeneous loading conditions with standardized tests for mechanical characterization, such as simple tension, equibiaxial extension and pure shear. Ogden’s model shares this beneficial property with earlier developments of principal stretch-based formulations of the strain-energy density functions, such as those by Carmichael & Holdaway [6] and Valanis & Landel [7]. The latter proposed a strain-energy density function for incompressible isotropic hyperelastic materials as the sum of a single scalar function evaluated at the three principal stretches, a representation that has become known as the Valanis–Landel hypothesis. Ogden’s model (1.1) hence belongs to the group of Valanis–Landel-type materials.

The argument of simplicity may have lost significance relative to the argument of robust numerical implementation with the advent of computational finite element techniques. In fact, it is well known that the computation of derivatives of the eigenvalues with respect to strain tensors and thus the determination of tangent tensors pose their own challenges under generic non-homogeneous loading conditions when the principal stretches change order and travel through coalescing values. However, algorithmic solutions (see e.g. [8], §5.6), closed-form expressions for the tangent tensors [9–12] and invariant-based reformulations [13,14] or approximations [15] have helped to overcome these problems so that Ogden’s model can efficiently be used with finite elements and is available in various software packages for mechanical analysis.

In addition to the advantage of compact mathematical form, the use of principal stretches in general and the Valanis–Landel hypothesis in particular allows an interpretation of the model in terms of the molecular statistical theory of rubber elasticity, which was not in the scope of the developments by Ogden [1]. To this end, the three eigenvectors of the right stretch tensor and their corresponding eigenvalues can be understood as the referential direction and elongation of the end-to-end vectors of three ideal representative chains, respectively. In fact, this link to the three-chain model was already highlighted in [7] and later elaborated in [16,17]. Along these lines, it was shown that Ogden’s phenomenological model can be reinterpreted in terms of a non-affine three-chain model of non-Gaussian chains [16], based on a specific completion of four essential steps: (i) postulation of a generally nonlinear relation between the deformation of the chain end-to-end vector and the macroscopic deformation; (ii) a suitable representation of the free energy of a single chain with fixed end-to-end length; (iii) complementation of the free energy through contributions from topological constraints; and (iv) averaging of the chains’ free energy to obtain the energy of the network. Adopting this idea, we show in the present work that, by accomplishing these steps in a different way, one both recovers other well-known models of rubber elasticity and easily discovers new forms that could turn out to be suitable for describing the behaviour of hyperelastic rubber-like materials. The schematic view furthermore allows Ogden’s model to be categorized and located within the large group of isotropic hyperelastic constitutive models of finite elasticity. Finally, by changing each of the four individual steps, we derive ‘variations’ on Ogden’s model and illustrate the performance of these ‘close relatives’ through application to Treloar’s experimental data on vulcanized rubber [18].

## 2. A route from statistical to continuum mechanical models

The free energy ψ=e−Θs of a single polymer chain in a rubber-like network at constant temperature Θ depends on the chain’s end-to-end distance r. Since changes of the internal energy e with r are assumed to be small relative to changes of the configurational entropy s, e is typically considered constant (see e.g. [19]) and hence does not contribute to the change in free energy due to network deformation, so that the relevant result is ψ∝−Θs. The entropy is affected not only by the end-to-end distance r of a test chain itself but also by the constraining effect of the other chains in the neighbourhood, which restrict the number of conformations that the test chain can take; see e.g. the review by Dal *et al.* [20]. A convenient, albeit clearly not exclusive, way of accounting for the latter effect is to visualize a constraining ‘tube’ [21–24] whose cross-section area scales with $d^{2}$. With respect to a reference state, the geometric quantities

2.1 $$r=\lambda_{r}r_{0}\;\mathrm{and}\;d^{2}=\frac{d_{0}^{2}}{\nu_{r}}$$

can be expressed through their referential values $r_{0}$ and $d_{0}$ in terms of two microkinematic variables [25], *viz*. the chain stretch $\lambda_{r}$ and a ‘tube contraction’ factor $\nu_{r}$ that relates to the change in cross-section area of the tube. Accordingly, the change in free energy Δψ of the test chain due to changes in the end-to-end length and tube diameter with respect to a fixed reference state can be represented as a function of the two microkinematic variables in the additive form

2.2 $$\Delta \psi =-\Theta \Delta s={\hat{\psi }}_{\lambda }(\lambda_{r})+{\hat{\psi }}_{\nu }(\nu_{r}),$$

which defines the contributions to the change in free energy from a reference state to a current state characterized through $\lambda_{r}$ and $\nu_{r}$, due to entropy changes caused by ‘chain stretching’ $\psi_{\lambda }={\hat{\psi }}_{\lambda }(\lambda_{r})$ and ‘tube contraction’ $\psi_{\nu }={\hat{\psi }}_{\nu }(\nu_{r})$, respectively [25]. The free energy per unit volume of the cross-linked chain network, i.e. the rubber-like material, is thus obtained as an average ⟨⋅⟩ over a suitable representative set of chains, multiplied by the chain density n, so that (cf. e.g. [25])

2.3 $$\Psi =\Psi_{\lambda }+\Psi_{\nu }=n(\langle \psi_{\lambda }\rangle +\langle \psi_{\nu }\rangle ),$$

where the network has been considered to be ideal, i.e. imperfections or loose-end effects [26] have been neglected.

Assuming that the chain network under consideration is large enough to obey the laws of continuum mechanics, its current state of deformation is related to the motion χ(X,t) at time t and is locally defined (to the first order) through the deformation gradient F(X,t)=Grad χ(X,t) at referential position X, where the arguments of F will be omitted in writing for the sake of brevity.

The free-energy equivalence (2.3) embodies a relation between the microscopic statistical realm and the macroscopic continuum mechanical world, which manifests itself in the averaging operation. To close the problem, further relations between microscopic and macroscopic scales are required which relate the kinematics on the two length scales. With the chosen microkinematic variables in (2.1), and restricting to elastic behaviour, this poses the problem of defining relations

2.4 $$\text{F}\mapsto \lambda_{r}\;\mathrm{and}\;\text{F}\mapsto \nu_{r}$$

for each single chain considered. Once these relations are established, their consideration in equation (2.3) allows the free energy per unit reference volume of the network to be rewritten in terms of F, and the arguments of material objectivity finally lead to the reduced representations in terms of the right stretch or Cauchy–Green tensors U and C, respectively [3,27], so that

2.5 $$\Psi =\hat{\Psi }(\text{F})=\overset{ˇ}{\Psi }(\text{U})=\hat{\Psi }(\text{C}),$$

where additional constraints may apply, in particular incompressibility, expressed for instance through the constraint function Γ(C)=detC−1=0 [28].

Equations (2.2)–(2.4) contain all four steps proposed in the introduction. Step (i) is contained in equation (2.4)${\;}_{1}$, step (ii) in equation (2.2), step (iii) in equations (2.4)${\;}_{2}$ and (2.2), and step (iv) in the average (2.3). In the next section, we briefly review the particular realizations of these steps that lead to Ogden’s model, as suggested in [16].

## 3. Four steps from long-chain molecules to Ogden’s model

We consider a set of N rigid links of length ℓ forming a freely jointed chain with fully extended length L=Nℓ between two cross-links in the network that forms a rubber-like material, which contains a number n of such chains per unit reference volume.

### (a) Power laws as the concept of non-affinity (i)

Let $R=r_{0}M$ denote the end-to-end vector of a chain, where M is a unit vector. When the network deforms, R changes to r=rm, with |m|=1. Under the affine assumption, the transformation results from the linear mapping R↦FR so that

3.1 $$\lambda_{r}=\frac{r}{r_{0}}=|\text{F}M|=\sqrt{M\cdot {\text{F}}^{T}\text{F}M}=\sqrt{M\cdot \text{C}M}=\lambda_{M}$$

equals the affine stretch $\lambda_{M}=|FM|$. Typically, however, the end-to-end vector cannot be regarded as a material line element of the continuum but rather as a member of a network whose current configuration is a result of minimizing the potential energy under the given kinematic constraints and boundary conditions [29–31]. Nowadays, computational models allow computation of the chain deformations and the statistics of their end-to-end vectors for large ensembles of chain models; see e.g. [30]. Notwithstanding, the affine assumption has also been replaced by more general formulations in continuum models, which may serve as better approximations of the chain deformation than the affine mapping. Fried [32], for example, considered mappings of the form R↦K(F)R, with det(K(F))>0 for all F with positive determinant. These mappings can generally represent nonlinear tensor-valued functions of F, and a particular choice is $K(F)=RU^{\beta }$ [16], where β is a real number and $R={FU}^{-1}$ is a (proper) orthogonal tensor resulting from the polar decomposition. The chain stretch thus becomes

3.2 $$\lambda_{r}=\frac{r}{r_{0}}=\frac{r_{0}|\text{R}{\text{U}}^{\beta }M|}{r_{0}}=|\text{R}{\text{U}}^{\beta }M|=\sqrt{M\cdot {\text{U}}^{2\beta }M}$$

and includes the affine approach (3.1) for β=1. An alternative relaxation of the affine assumption is obtained by assuming the chain stretch to be a real power of the affine stretch (3.1) (e.g. [33,34]),

3.3 $$\lambda_{r}=\lambda_{M}^{\beta }={\left(\sqrt{M\cdot \text{C}M}\right)}^{\beta }.$$

Amores *et al.* [35] recently proposed to define the chain stretch by the projection of the right stretch tensor as $\lambda_{r}=M\cdot UM$, and the *ad hoc* generalization, which contains the original relation [35] as a special case (β=1), reads

3.4 $$\lambda_{r}=M\cdot {\text{U}}^{\beta }M.$$

Moreover, introducing a r-norm-like operation with r≥1 a real number, the latter expressions can be unified as

3.5 $$\lambda_{r}={\left(M\cdot {\text{U}}^{\beta r}M\right)}^{1/r}.$$

In fact, relation (3.5) includes the models (3.2)–(3.4) and the one in [35] for r=2, r=2/β, r=1 and r=β=1, respectively, which differ in the general case of arbitrary M. However, for chains oriented along the Lagrangian principal directions, i.e. for $M=N_{k}$ with k=1,2,3 referring to the eigenvectors of U, the relations (3.2)–(3.5) coincide independently of the values of β and r, and give

**Figure 1.**
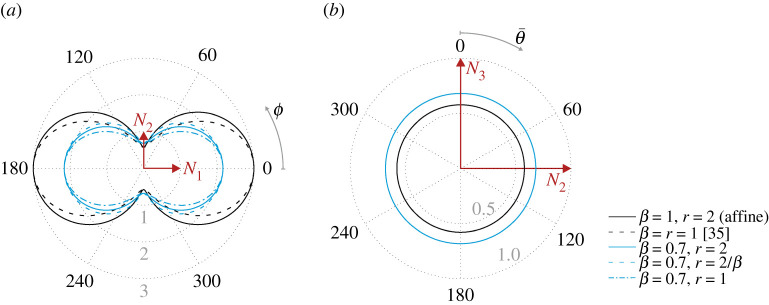
Spherical plots of the chain stretch for each reference orientation in the $N_{1}\text{–}N_{2}$ plane (*a*) and $N_{2}\text{–}N_{3}$ plane (*b*). The angle variable $\bar{\theta }$ is an extension of θ to the interval 0<ϕ≤2π, in order to uniquely represent all unit vectors in the ϕ=π/2 plane with a single variable. Note that the   scale in (*b*) is three times larger than that in (*a*). (Online version in colour.)

3.6 $$\lambda_{r}\mapsto \lambda_{k}^{\beta }=|\text{F}N_{k}{|}^{\beta }$$

for any of the three directions $N_{k}$. Any of these four non-affine definitions of the chain stretch could thus form the basis of a molecular statistical interpretation of Ogden’s model provided in [16]. In figure 1, we illustrate the dependence of the predicted non-affine stretch for some values of β and r in relation (3.5). Expressing the unit vector M in terms of spherical coordinates 0≤ϕ<2π and 0≤θ<π, i.e.

3.7 $$M=\cos\phi \sin\theta N_{1}+\sin\phi \sin\theta N_{2}+\cos\theta N_{3},$$

$\lambda_{r}$ can be represented in a spherical plot, whose cross-sections for θ=π/2 and ϕ=π/2 are shown in figure 1 for uniaxial extension by a factor of 3 along $N_{1}$ with volume-preserving lateral contraction. As stated before, along the principal directions (identified by ϕ and θ equal to {0,π/2} in the plots), the stretch value depends only on β and is independent of r.

### (b) Power series as the representation of non-Gaussian chain statistics (ii)

Next, we consider the free-energy contribution $\psi_{\lambda }$ due to the configurational entropy s of a chain. Given the force f acting at the ends of a chain separated by a distance r, the corresponding change in free energy from a reference state ($\lambda =r/(\sqrt{N}\ell )=1$) in terms of the chain stretch reads (cf. e.g. §3.8 in [19])

3.8 $$\psi_{\lambda }=\int_{1}^{\lambda_{r}}f(\lambda )\sqrt{N}\;\ell \;d\lambda .$$

Even if only an approximation of the exact solution itself, the non-Gaussian statistical models based on the inverse Langevin function $L^{-1}(x)$ for 0≤x<1 [19] are most commonly used. For these models, the force reads $f(\lambda )=k_{B}\Theta /\ell \;L^{-1}(\lambda /\sqrt{N})$ (cf. eqn 6.10 in [19]), and equation (3.8) becomes

3.9 $$\psi_{\lambda }=\sqrt{N}\;k_{B}\Theta \;\int_{1}^{\lambda_{r}}L^{-1}\left(\frac{\lambda }{\sqrt{N}}\right)d\lambda ,$$

where $k_{B}$ and Θ denote Boltzmann’s constant and the absolute temperature, respectively. Since the inverse Langevin function cannot be given in closed form, it is typically approximated through either Taylor series or rational approximants (see §4), the latter of which are able to preserve the asymptotic behaviour of the inverse Langevin function $L^{-1}(x)$ at the extensibility limit x=1 [36,37]. While Taylor expansions lack this property [36,37], they can provide very accurate approximations within the stretch range covered by the convergence radius [38].

Moreover, it has been shown in [16] that remarkably close approximations of $L^{-1}(x)$ in the range of x values most relevant for modelling, i.e. distant from x=0 and x=1, can also be obtained through a weighted sum of power functions with $R^{+}$ terms of the form

3.10 $$L^{-1}(x)\approx G_{R}(x)=\sum_{i=1}^{R^{+}}m_{i}\;x^{a_{i}}$$

with all positive coefficients $m_{i}>0$ and powers $a_{i}>0$, already for the $R^{+}=2$ and $R^{+}=3$ terms. Insertion of this ‘generalized power series’ into (3.9) leads to the chain free energy

3.11 $$\psi_{\lambda }(\lambda_{r})=k_{B}\Theta \sum_{i=1}^{R^{+}}\frac{M_{i}}{A_{i}}(\lambda_{r}^{A_{i}}-1)$$

with coefficients and powers

3.12 $$M_{i}=N^{(1-a_{i})/2}m_{i}\;\text{and}\;A_{i}=a_{i}+1,$$

respectively. Although from a formal point of view one might prefer using Taylor series or rational approximants to capture the characteristics of the inverse Langevin function for either small x or values close to x=1, respectively, the formal similarity of (3.11) to the strain-energy density function of Ogden’s model (1.1) obtained through use of the approximation (3.10) is evident.

### (c) Power series expression for the tube contraction (iii)

Different constraints have been considered that restrict the configurations of a test chain in the network and reflect the mutual interaction and entanglements between the chains as well as effects of excluded volume; see e.g. [39]. In particular, the tube model [21,22] has been successfully included in various applied models of rubber elasticity, such as those in [25,40–42]. An implementation of the tube constraint by Miehe [25] postulates the free-energy change $\psi_{\nu }$ of a chain due to the spatial constraints as a linear function of the tube contraction. With slight changes in notation, this reads (cf. [25])

3.13 $${\hat{\psi }}_{\nu }(\nu_{r})=k_{B}\Theta w_{\nu }\Delta \nu_{r}=k_{B}\Theta w_{\nu }(\nu_{r}-1),$$

where the parameter $w_{\nu }$ takes into account the size and geometry of the chain and tube [16,22,25]. Kinematic considerations suggest that the tube diameter changes with the change in area $\zeta_{M}$ of surface elements perpendicular to the chain’s end-to-end vector, which is expressed through Nanson’s formula

3.14 $$\zeta_{M}=|\mathrm{cof}\;\text{F}\;M|=\sqrt{M\cdot (\det\text{C}){\text{C}}^{-1}M}$$

and can be understood as the tube contraction according to the affine model. A general, albeit phenomenological, relation between the area change (3.14) and the tube contraction $\nu_{r}$ is obtained through a sum of power functions of the form [16]

3.15 $$\nu_{r}=\sum_{j=1}^{R^{-}}\frac{{\bar{m}}_{j}}{{\bar{a}}_{j}}\;\zeta_{M}^{{\bar{a}}_{j}},$$

which generalizes the power-law expression suggested in [25]. The definition of the tube contraction (2.1)${\;}_{2}$ suggests that the powers ${\bar{a}}_{j}$ and coefficients ${\bar{m}}_{j}$ are positive throughout to reflect the increasing tube diameter with decreasing surface area [25]. Moreover, defining the reference state through $\nu_{r}=1$, which implies the normalization condition $\sum_{j=1}^{R^{-}}{\bar{m}}_{j}/{\bar{a}}_{j}=1$, equation (3.15) specifies (3.13) to

3.16 $${\hat{\psi }}_{\nu }(\nu_{r})=k_{B}\Theta w_{\nu }\sum_{j=1}^{R^{-}}\frac{{\bar{m}}_{j}}{{\bar{a}}_{j}}(\zeta_{M}^{{\bar{a}}_{j}}-1).$$

It is worth noting that if M is aligned with one of the three principal directions of strain, i.e. $M=N_{k}$ for k=1,2,3, the areal stretch results in $\zeta_{N_{k}}=\lambda_{i}\lambda_{j}$ for k≠i≠j≠k, which reduces to $\lambda_{k}^{-1}$ in the incompressible case. In this case, the one-term model ($R^{-}=1$) of (3.16) complies with the theory developed by Heinrich and co-authors [33,40,43].

### (d) Valanis–Landel hypothesis as the averaging operation (iv)

Any of the relations (3.2)–(3.5) or a more general relation (2.4)${\;}_{1}$ inserted into (3.11), together with (3.16), allows formulation of a model of the single chain’s change in entropy, and thus the free energy Δψ, as a result of a macroscopic network deformation F, so that $\Delta \psi =\tilde{\omega }(F,M)$. In order to obtain the corresponding free-energy density for a network with n chains per unit reference volume that contributes to the strain-energy density of the rubber-like material, a suitable average needs to be formulated according to (2.3), such as the arithmetic mean over a number M of representative chains with directions $M_{i}$ (cf. e.g. [16,25,44]),

3.17 $$\Psi =n\;\frac{1}{M}\sum_{i=1}^{M}\tilde{\omega }(\text{F},M_{i}).$$

The average over M=3 chains aligned with the three principal directions of strain $N_{k}$ may be seen as one particular realization of this average and has implicitly been used in the early developments of rubber-elasticity theory [45]. It has been noted [16,35] that this choice is in agreement with the additive decomposition suggested by Valanis & Landel [7],

3.18 $$\Psi =\hat{\Psi }(\text{C})=\tilde{\Psi }(\lambda_{1},\lambda_{2},\lambda_{3})=\omega (\lambda_{1})+\omega (\lambda_{2})+\omega (\lambda_{3}),$$

and similar arguments can be found in the original work [7]. In fact, this agreement holds if and only if the free energy of a chain aligned with $N_{k}$ is completely defined through the principal stretch $\lambda_{k}$ so that $\tilde{\omega }(F,N_{k})\propto \omega (\lambda_{k})$, and ω=nΔψ/3 in this case, in view of the relations (2.2) and (2.3).

### (e) Statistical representation of Ogden’s material parameters

The Valanis–Landel-type ‘average’ (3.18) over the three principal directions applied to the additive representation of the free energy (2.2), the non-affine chain stretch (3.5) considered in (3.11) and the constraint contribution (3.16) yields the reconciliated form of Ogden’s model [16]

3.19 $$\Psi =\frac{nk_{B}\Theta }{3}\sum_{k=1}^{3}\left[\sum_{i=1}^{R^{+}}\frac{M_{i}}{A_{i}}(\lambda_{k}^{\beta A_{i}}-1)+\sum_{j=1}^{R^{-}}\frac{w_{\nu }{\bar{m}}_{j}}{{\bar{a}}_{j}}(\lambda_{k}^{-{\bar{a}}_{j}}-1)\right],$$

which coincides with the original model (1.1) formulated by Ogden [1] through the definitions [16]

3.20 $$\mu_{r}=\frac{nk_{B}\Theta }{3}N^{(1-a_{r})/2}m_{r}\beta \;\text{and}\;\alpha_{r}=\beta (a_{r}+1)$$

for $1<r\leq R^{+}$, associated with the terms that have positive powers and coefficients, and

3.21 $$\mu_{r}=-\frac{nk_{B}\Theta }{3}w_{\nu }{\bar{m}}_{r-R^{+}}\;\text{and}\;\alpha_{r}=-{\bar{a}}_{r-R^{+}}$$

for $R^{+}<r\leq (R=R^{+}+R^{-})$, i.e. the negative terms. Note that $m_{r}$ and $a_{r}$, $r=1,2,\ldots ,R^{+}$, are not free material parameters but predefined ‘constants’, as they define the ‘power series' $G_{R}$ in (3.10) which approximates the inverse Langevin function [16].

## 4. Modelling scheme: examples

**Figure 2.**
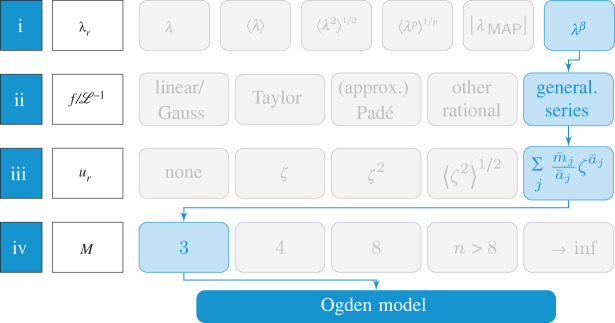
Graphical scheme of the steps (i)–(iv) in the route to deriving Ogden’s model. (Online version in colour.)

The interest spanning more than one century in modelling the mechanical behaviour of rubber-like materials has resulted in a wealth of constitutive models based both on statistical considerations and on empirical observations of the stress–strain behaviour of rubber parts and specimens under mechanical loads. A large amount of this work has focused on the large-strain elastic behaviour of these materials expressed in terms of the theory of hyperelasticity (see e.g. [3], §79), and comprehensive reviews can be found in [20,46–48]. Although the four steps [16] restated above to reconcile Ogden’s model with the molecular statistical theory may admittedly seem simplistic in view of the great body of work on the statistical mechanics of polymers, they nevertheless serve as a recipe to establish such a link for several other seemingly phenomenological models of rubber elasticity as well. To this end, the scheme shown in figure 2 is considered, which illustrates the steps performed in §3 to obtain Ogden’s model, together with alternatives to each of these steps.

The alternatives displayed in figure 2 reflect a representative choice and are clearly not exclusive. For step (i), for example, the scheme includes the affine stretch λ according to (3.1), where the index M has been omitted for the sake of brevity, the (simple) average of the affine stretch ⟨λ⟩ [49], the root mean of the affine square stretch $\sqrt{\left\langle \lambda^{2}\right\rangle }=\sqrt{\mathrm{tr}\;C/3}$ [50,51], the p-root averaged stretch [25], and the average stretch in the maximal advance path $\lambda_{\mathrm{MAP}}$ [52]. Examples of other choices for approximating the inverse Langevin function (ii) include its linearization, which agrees with the Gaussian chain model (see e.g. [19], §6), Taylor series expansions (e.g. [53–55]), and Padé approximants and adjusted versions of them or other rational functions (e.g. [17,19,36,37,56–58]). Noting that the inverse Langevin statistics represents an approximation to non-Gaussian chains itself, the inverse Langevin function may be replaced by the corresponding expressions for the force f that result from refined statistical approaches; see e.g. [59]. The tube contraction (2.1)${\;}_{2}$ giving rise to constraint contributions to the free energy (iii) can be either neglected or assumed to be related to the area change of surface elements ζ in (3.14) (e.g. [42]), powers thereof, i.e. $\zeta^{q}$ [25,33,43,60], or the root mean square $\sqrt{\left\langle \zeta^{2}\right\rangle }$ (cf. [16,59]), instead of the generalized representation (3.15). Finally, instead of three chains used to accomplish step (iv), four could be considered according to the tetrahedral model [61], the eight-chain concept could be employed [62], or an arbitrary number of distinct directions on the unit sphere may be used as an approximation of a full network with a continuous distribution of chains in all directions; see e.g. [10,63,64].

The scheme in figure 2 serves as a ‘model generator’, providing a molecular statistically motivated model for each combination of the four steps. An advantage of this approach to developing hyperelastic models for rubber-like materials is the physical interpretability of the associated material parameters, which are inherited from the assumptions within the different steps. This property may turn out to be beneficial when identifying these parameters in comparison with experimental data, since the physical meaning implies bounds and suggests reasonable ranges for the values that these constants can take; see e.g. [46]. Before using the scheme (figure 2) in §5 to analyse variations from the path indicated in figure 2, the next section will review some known routes through the scheme and illustrate that, by combining appropriately some of the alternative steps, one arrives at familiar hyperelastic models of rubber elasticity.

### (a) $I_{1}$-based models

Several of the established phenomenological models based on the first principal invariant

4.1 $$I_{1}=\mathrm{tr}\;\text{C}$$

allow an interpretation in terms of statistical mechanics by comparison with the Arruda–Boyce model [62], which was proposed in view of the statistical nature of rubber elasticity and thus contains only constants with physical interpretations. The corresponding strain-energy density [62] is obtained by starting from the affine stretch assumption, approximating the inverse Langevin function through a truncated Taylor series [54], omitting contributions from topological constraints, and choosing M=8 chains that span from the centre to the corners of a regular cube which deforms into a cuboid co-aligned with the principal axes of strain. A striking result of this arrangement of chains is that they feature the same stretch [62]

4.2 $$\lambda_{r}=\sqrt{\frac{I_{1}}{3}},$$

which is uniquely expressed in terms of the first principal invariant $I_{1}=\mathrm{tr}\;C$. Upon integration of the Taylor series, the first three terms of the strain-energy function read [62]

4.3 $$\Psi_{\mathrm{AB}}=nk_{B}\Theta \left[\frac{1}{2}(I_{1}-3)+\frac{1}{20N}(I_{1}^{2}-9)+\frac{11}{1050N^{2}}(I_{1}^{3}-27)+\ldots \right]$$

and evidently recover Yeoh’s model [65]

4.4 $$\begin{array}{rl} \Psi_{\mathrm{Yeoh}} & \;=C_{1}(I_{1}-3)+C_{2}{\left(I_{1}-3\right)}^{2}+C_{3}{\left(I_{1}-3\right)}^{3}\\ & \;=(C_{1}-6C_{2}+27C_{3})(I_{1}-3)+(C_{2}-9C_{3})(I_{1}^{2}-9)+C_{3}(I_{1}^{3}-27), \end{array}$$

with a special choice of the parameters $C_{1}$, $C_{2}$ and $C_{3}$ expressed in terms of the chain and network parameters N, $k_{B}$, Θ and n, and found by comparison of (4.4) with (4.3). Similar considerations for the full series render the Arruda–Boyce model a special case of Rivlin’s generalized invariant representation [66] with all terms dependent on the second principal invariant set to zero ($C_{00}=0$ and $C_{ij}=0$ for all j≠0), i.e.

**Figure 3.**
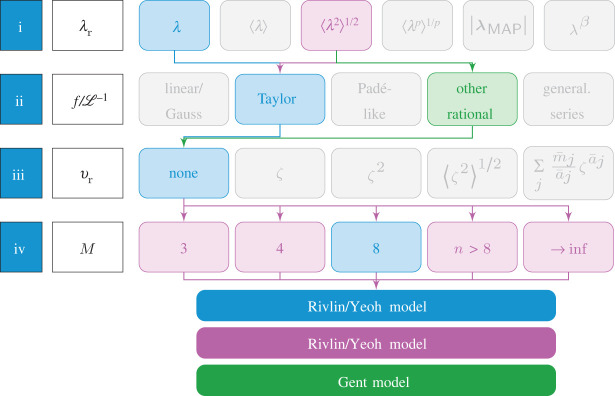
Graphical scheme of the steps (i)–(iv) with indicated routes to deriving the Rivlin, Yeoh and Gent models. (Online version in colour.)

4.5 $$\Psi_{R}=\sum_{i=1}^{\infty }\sum_{j=1}^{\infty }C_{ij}{\left(I_{1}-3\right)}^{i}{\left(I_{2}-3\right)}^{j}\equiv \sum_{i=1}^{\infty }C_{i0}{\left(I_{1}-3\right)}^{i}=\sum_{i=1}^{\infty }{\bar{C}}_{i0}(I_{1}^{i}-3),$$

which clearly includes (4.4) upon truncation of the series. Moreover, Beatty’s reinterpretation of the Arruda–Boyce model as an average stretch model [51] with $\lambda_{r}=\sqrt{\left\langle \lambda^{2}\right\rangle }=\sqrt{I_{1}/3}$, for which in fact the number of representative chains M becomes irrelevant, implies that more than one ‘route’ within the proposed four-step scheme can lead to the same phenomenological model, as indicated by the purple path in figure 3.

Apart from $I_{1}$-polynomial-type free energies, known to be inadequate for describing the chain extensibility limit [37], the same strategies highlighted in blue and purple in figure 3 yield Gent’s model [67,68] with parameters $\mu_{G}$ and $J_{m},$

4.6 $$\Psi_{\mathrm{Gent}}=-\frac{\mu_{G}J_{m}}{2}\ln\left[1-\frac{I_{1}-3}{J_{m}}\right],$$

if instead of a Taylor series the inverse Langevin function is approximated through a rational expression of the form $L^{-1}(x)\approx 3x/(1-x^{2})$, in line with the approach of Warner [56]; see also [58]. This is indicated by the green path in figure 3. Note that this rational function is not a ‘mathematically strict’ approximant, and in particular not a Padé approximant of $L^{-1}$ [37]. However, it correctly reproduces the asymptotic behaviour as x→1 [69], and it captures the Gaussian limit $L^{-1}\approx 3x$ as x≪1. Integration of (3.9) yields

4.7 $$\psi_{\lambda }=k_{B}\Theta \sqrt{N}\int_{1}^{\sqrt{I_{1}/3}}\frac{3\lambda /\sqrt{N}}{1-\lambda^{2}/N}\;d\lambda =-\frac{3}{2}k_{B}\Theta N\ln\left[1-\frac{I_{1}-3}{3(N-1)}\right],$$

and without the contribution from topological constraints direct comparison of the remaining network free energy $\Psi =n\psi_{\lambda }$ with (4.6) finally specifies the material parameters in Gent’s model,

4.8 $$\mu_{G}=nk_{B}\Theta \frac{N}{N-1}\;\text{and}\;J_{m}=3(N-1),$$

in terms of statistical parameters. This close relation between the Gent model and the molecular statistical theory is well known and was discussed in [17,70] and also in [37] with a slightly different interpretation of the factor N/(N−1), which was associated with the approximation of $L^{-1}(x)$ in [37], whereas in (4.8) it changes the meaning of the parameter $\mu_{G}$ in Gent’s model to a modified shear modulus. Nevertheless, one notes that for large N the factor approaches 1 [37] and this distinction is of little relevance. Other rational function approximations that capture the asymptotic behaviour as x→1, as reviewed in [58] for example, may lead to other ‘limited elastic’ $I_{1}$-materials [69] with similar behaviour.

### (b) Models that include $I_{2}$

Already early in the development of the continuum mechanical theories of rubber elasticity, the deviations between the $I_{1}$-dependent neo-Hookean model obtained from the Gaussian statistics and experimental data [71–73] provided motivation to include terms that depend on the second principal invariant,

4.9 $$I_{2}=\mathrm{tr}({\text{C}}^{-1}\det\text{C})=\frac{1}{2}[{\left(\mathrm{tr}\;\text{C}\right)}^{2}-\mathrm{tr}\;{\text{C}}^{2}],$$

in the strain-energy density function [74], such as in the Mooney–Rivlin model [75,76]. Explanations of $I_{2}$-contributions in terms of molecular statistics were provided in terms of non-Gaussian chain behaviour [72], as well as through restricting effects on the number of chain configurations caused by neighbouring chains [73]. The latter effect was also associated with non-affine deformation of the chains [77], and Fried [32] achieved $I_{2}$-dependent terms by use of a non-affine relaxation of the chain stretch, i.e. a particular choice of step (i).

The confining effect of neighbouring chains is also at the basis of the tube constraint (§2). Similarly to how the unique expression of the chain stretch $\lambda_{r}=\sqrt{I_{1}/3}$ in the eight-chain (or average stretch) approach (4.2) paves the way for statistical interpretations of models that exclusively depend on the first principal invariant, consideration of Kearsley’s result [50,51] $\sqrt{\left\langle \zeta^{2}\right\rangle }=\sqrt{I_{2}/3}$ allows one to relate the tube contraction to macroscopic deformation, as discussed in [16,78] and implemented, for instance, in [42,59]. By this choice, the affine chain stretch (i), linearization of $L^{-1}(x)$ (ii), $\nu_{r}=\zeta_{M}^{2}$ (iii) and choosing M=8 chains along the diagonals of the cuboid deforming with the principal directions (iv), as in the Arruda–Boyce approach, lead to

**Figure 4.**
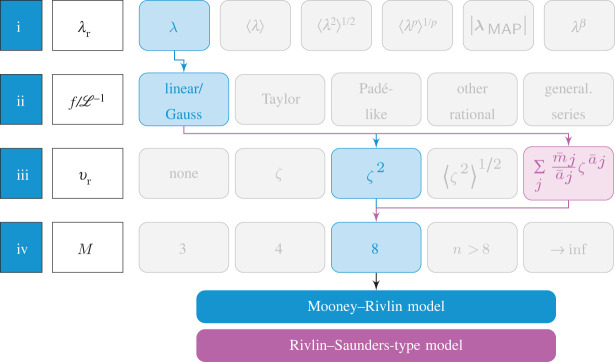
Graphical scheme of the steps (i)–(iv) with the routes to deriving Mooney–Rivlin and Rivlin–Saunders-type models. (Online version in colour.)

4.10 $$\Psi =\frac{nk_{B}\Theta }{6}[3(I_{1}-3)+2w_{\nu }(I_{2}-3)]=c_{1}(I_{1}-3)+c_{2}(I_{2}-3),$$

which is the Mooney–Rivlin model [75,76] with $c_{1}=(nk_{B}\Theta )/2$ and $c_{2}=(nk_{B}\Theta w_{\nu })/3$ (cf. figure 4).

Moreover, a special form of the generalized series in step (iii) in figure 4 with ${\bar{a}}_{j}=2j$ and ${\bar{m}}_{j}/{\bar{a}}_{j}=c_{j}$ leads to a power series, i.e.

4.11 $$\nu_{r}=\sum_{j=1}c_{j}\;\zeta^{2j}\;\mathrm{where}\;\sum_{j=1}c_{j}=1$$

with even powers and scalar coefficients $c_{j}$ for j=1,2,…. If (4.11) converges to the analytic function $g(\zeta^{2})$, the network free energy obtained through the M=8 chains can be represented in the Rivlin–Saunders form

4.12 $$\Psi_{\mathrm{RS}}=c_{1}(I_{1}-3)+f(I_{2}-3)=\frac{nk_{B}\Theta }{2}[(I_{1}-3)+2w_{\nu }[g(I_{2})-1]]$$

and allows the interpretation of the $f(I_{2}-3)$-contribution in the Rivlin–Saunders theory [79] as an indicator of how the tube diameter changes with macroscopic deformation.

### (c) A family of models

The scheme presented in figures 2–4 and, as a particular example, the relationship between many invariant-based models, which is revealed in their interpretation in terms of ingredients from the molecular statistical theory, serve to illustrate the idea of ‘close and distant relatives’ among them. The four-step procedure establishes a family of models that can all be shown to originate from the same ingredients of the statistical mechanics of long-chain molecules, regardless of whether this was part of their original development or not. This makes them at least distant relatives. It is evident that for all phenomenological members of this family this implies—possibly strong—restrictions on the meaningful range of values that their parameters can take. The representation of Yeoh’s model (4.4) exemplifies this, since n and N now become the only two ‘degrees of freedom’ for fitting the model to experimental data, compared to the three parameters $C_{1},C_{2}\text{ and }C_{3}$. However, one might argue that if the phenomenological constants provide an acceptable match with experimental data on rubber, they should capture the underlying physics and thus imply reasonable values for the statistical parameters. In fact, for Ogden’s model, it was shown that the fitted parameters $\mu_{r}$ and $\alpha_{r}$ indeed lead to favourable agreement between the series (3.10) and the inverse Langevin function [16].

More members of this family may be identified by travelling new paths within the scheme, or by increasing the length of each row that represents a step, where both existing approaches not mentioned here and future developments may serve this purpose. The greatest potential in this regard is associated with the contribution from topological effects, which was here restricted only to a simple implementation of the confining tube concept [21,22], and even more limited to relations between the change of surface elements (3.14) and the tube diameter d. Although the elaboration of this concept already encompasses a broad spectrum of models, the ‘tube contraction’ [25] itself (equation (2.1)${\;}_{2}$) would generally allow for a more general dependence on F than is contained in (3.15), potentially giving rise to further expressions that comply with those phenomenological models, and particularly to terms that include both $I_{1}$ and $I_{2}$. Darabi & Itskov [34], for example, recently proposed a model for the constraint contribution that combines ideas from the tube and slip-link [80] theories. The corresponding expression for the free energy (eqn 27 in [34]) cannot be brought in the form (3.15) with only positive constants.

In light of the previous considerations, the known proximity of the Arruda–Boyce and Gent models is based on the fact that they differ only in the representation of the chain force, i.e. a single variation within the four-step procedure, which allows them to be distinguished from each other. Such single variations lead to what we will refer to as ‘close relatives’ and which we will analyse in the next section with respect to Ogden’s model in a molecular statistical form (3.19) as a basis.

## 5. Variations on Ogden’s model

The incompressible Ogden model [1] in its unaltered original form is indisputably one of the most successful and versatile hyperelastic approaches to studying rubber elasticity, with many uses even beyond this class of materials. Notwithstanding, a study of some of its close relatives will provide a new perspective on this important milestone of phenomenological rubber-elasticity theory, and moreover indicate a few other models with similar capacities to the original.

### (a) Variation 1: chain model

First, we will study the variation of step (ii), the expression that provides the force f at the ends of a chain extended by a factor $\lambda_{k}$, and which finally defines the free energy $\psi_{\lambda }$, whereas all other steps follow §3. By this means, the free energy of the chain network (2.3) takes the general form

5.1 $$\Psi =n\frac{1}{3}\sum_{k=1}^{3}\left[{\hat{\psi }}_{\lambda }(\lambda_{k}^{\beta })+k_{B}\Theta \sum_{j=1}^{R^{-}}\frac{{\bar{m}}_{j}^{\prime }}{{\bar{a}}_{j}}(\lambda_{k}^{-{\bar{a}}_{j}}-1)\right],$$

where the abbreviation ${\bar{m}}_{q}^{\prime }=w_{\nu }{\bar{m}}_{q}$ has been introduced [16]. Exemplarily, we will derive the non-affine three-chain models that are close relatives of the original model and are obtained by consideration of Taylor series or rational approximants for the inverse Langevin function, as well as an alternative representation of non-Gaussian chain statistics.

#### (i) Taylor series and Gaussian chains

Expressing the inverse Langevin function in terms of a Taylor series approximation [19,54] leads to strain-energy functions of the Ogden-model family

5.2 $${\hat{\psi }}_{\lambda }(\lambda_{k}^{\beta })=k_{B}\Theta \left(\frac{3}{2}(\lambda_{k}^{2\beta }-1)+\frac{9}{20N}(\lambda_{k}^{4\beta }-1)+\frac{297}{1050N^{2}}(\lambda_{k}^{6\beta }-1)+\cdots \right)$$

with interpretations of the parameters $\mu_{i}$ and $\alpha_{i}$, $i=1,2,\ldots ,R^{+}$, as

5.3 $$\mu_{i}=\frac{3nk_{B}\Theta }{6},\frac{9nk_{B}\Theta }{60N},\frac{297nk_{B}\Theta }{3150N^{2}},\ldots \;\text{and}\;\alpha_{i}=2\beta ,4\beta ,6\beta ,\ldots .$$

For example, the Gaussian limit, i.e. the first term in (5.2), gives the free energy

5.4 Ψ=nkBΘ3∑k=13[32(λk2β−1)+∑j=1R−m¯j′a¯j(λk−a¯j−1)].

In general, these expressions do not have any advantage over the more general representation (3.20). Nevertheless, they have an interesting implication: in fact, they suggest that one could approximate the inverse Langevin function $L^{-1}(x)$ within a range of interest in terms of a weighted sum of powers which are odd multiples of 1/β, i.e. $\sum_{i}w_{i}x^{(2i-1)/\beta }$, so as to obtain an Ogden-type model with only integer powers. Such a model would bring advantages in terms of the numerical treatment, since its strain-energy function could be represented in terms of principal traces of C; see also [15].

#### (ii) Rational approximants of $L^{-1}$

Further close relatives are obtained by consideration of some of the various rational function approximations proposed for $L^{-1}$. For example, using Cohen’s rounded Padé approximant [81]

5.5 $$L^{-1}(x)=x\frac{3-x^{2}}{1-x^{2}}$$

yields the network free energy

5.6 Ψ=nkBΘ3∑k=13[λk2β−12−Nln⁡(λk2β−N1−N)+∑j=1R−m¯j′a¯j(λk−a¯j−1)],

and for later use we also provide the corresponding principal nominal stresses

5.7 $$P_{k}=\frac{nk_{B}\Theta }{3}\left[\beta \lambda_{k}^{2\beta -1}\frac{3-\lambda_{k}^{2\beta }/N}{1-\lambda_{k}^{2\beta }/N}-\sum_{j=1}^{R^{-}}{\bar{m}}_{j}^{\prime }\lambda_{k}^{-{\bar{a}}_{j}-1}\right]-p\lambda_{k}^{-1},\;k=1,2,3.$$

Using more recent rational approximations (see e.g. [58]) clearly leads to various alternative forms of the strain-energy function.

#### (iii) Alternative approximation of non-Gaussian chains

Khiêm & Itskov [59] recently deviated from the Langevin statistical approach and used another approximation to the non-Gaussian statistics of freely jointed chains, based on the model proposed by Ilg [82]. In terms of the force f at the ends of a freely jointed chain with N links of length ℓ, stretched by the factor λ, the model gives (cf. [59,82])

5.8 $$f=\frac{9k_{B}\Theta }{\ell \pi^{2}}\left(\frac{\sqrt{N}}{\lambda }-\pi \cot\frac{\pi \lambda }{\sqrt{N}}\right).$$

By integration, the free energy (3.8) follows as

5.9 Ψ=nkBΘ3∑k=13[9Nπ2ln⁡(λkβsin⁡(π/N)sin⁡(πλkβ/N))+∑j=1R−m¯j′a¯j(λk−a¯j−1)],

and the principal stresses read

5.10 $$P_{k}=\frac{nk_{B}\Theta }{3}\left[\frac{9\sqrt{N}}{\pi^{2}}\beta \lambda_{k}^{\beta -1}\left[\frac{\sqrt{N}}{\lambda_{k}^{\beta }}-\pi \cot\left(\frac{\pi \lambda_{k}^{\beta }}{\sqrt{N}}\right)\right]-\sum_{j=1}^{R^{-}}{\bar{m}}_{j}^{\prime }\lambda_{k}^{-{\bar{a}}_{j}-1}\right]-p\lambda_{k}^{-1},$$

where k=1,2,3.

### (b) Variation 2: representative chains

The second variation that we consider concerns the number of representative chains, i.e. a modification of the three-chain concept, which is implicit in the Valanis–Landel hypothesis.

#### (i) Full-network model

When considering a large number of chains with normalized end-to-end vectors $M_{l}$, l=1,2,…,M, suitably distributed on the unit sphere as a discrete approximation of the full-network model (see e.g. [83]), the distinction between the non-affine relations (3.2)–(3.5) becomes relevant, whereas they all agree for three chain vectors aligned with the principal directions. We limit our study to the full-network analogue obtained from relation (3.5), which leads to the simplest expressions for the stress tensor. Under consideration of incompressibility (detC=1) the microkinematic variables become, in this case,

5.11 $$\lambda_{r}\mapsto \lambda_{M_{l}}^{\beta }={\left(\sqrt{M_{l}\cdot \text{C}M_{l}}\right)}^{\beta }\;\text{and}\;\nu_{r}\mapsto \nu_{M_{l}}=\sqrt{M_{l}\cdot {\text{C}}^{-1}M_{l}}$$

for l=1,2,…,M, i.e. powers of the affine stretch $\lambda_{M}$ and the affine change in area of surface elements perpendicular to M. The corresponding discrete full-network model reads

5.12 Ψ=nkBΘM∑l=1M[∑r=1R+MrAr(λMlβAr−1)+∑j=1R−m¯j′a¯j(νMla¯j−1)]

and is distinguished from (3.19) only through the M directions considered. The first Piola–Kirchhoff stresses in the principal directions can be obtained by means of the projections of $S=2\partial \Psi /\partial C-pC^{-1}$ onto $N_{k}$ and multiplication with $\lambda_{k}$ as

5.13 $$\begin{array}{rl} & P_{k}=\lambda_{k}\frac{nk_{B}\Theta }{M}\sum_{l=1}^{M}\left[\sum_{r=1}^{R^{+}}N^{(1-a_{r})/2}m_{r}\beta \lambda_{M_{l}}^{\beta (a_{r}+1)-2}{\left(N_{k}\cdot M_{l}\right)}^{2}-\sum_{r=1}^{R^{-}}{\bar{m}}_{j}^{\prime }\nu_{M_{l}}^{{\bar{a}}_{j}-2}{\left(N_{k}\cdot {\text{C}}^{-1}M_{l}\right)}^{2}\right]-p\lambda_{k}^{-1}, \end{array}$$

where k=1,2,3. We note that while ${\bar{a}}_{1}>1$ was noted to be sufficient to guarantee material stability of the model response for the three-chain representation (3.19), arguments of convex composition (see e.g. Lemma B.9 in [84]) can be used to infer that ${\bar{a}}_{1}>2$ guarantees convexity of the strain energy with respect to $M_{l}\cdot (C^{-1}\det C)M_{l}$ [84–86].

#### (ii) Eight-chain models

An eight-chain variant of Ogden’s model follows from a special case of (5.12) by consideration of M=8 chains with normalized end-to-end vectors that point to the eight corners of a cube that deforms and aligns with the principal axes according to [62]. The stretches and tube contractions, respectively, of all the eight directions coincide, and consideration of (5.11) yields

5.14 $$\lambda_{r}={\left(\frac{I_{1}}{3}\right)}^{\beta /2}\;\mathrm{and}\;\nu_{r}={\left(\frac{I_{2}}{3}\right)}^{1/2},$$

so that the network free energy reads

5.15 Ψ=nkBΘ[∑r=1R+MrAr[(I13)βAr/2−1]+∑j=1R−m¯j′a¯j[(I23)a¯j/2−1]].

Upon collecting and renaming the constants, (5.15) can be represented as

5.16 $$\Psi =\sum_{i=1}^{R^{+}}C_{i}\left[{\left(\frac{I_{1}}{3}\right)}^{\gamma_{i}}-1\right]+\sum_{j=1}^{R^{-}}D_{j}\left[{\left(\frac{I_{2}}{3}\right)}^{\delta_{j}}-1\right].$$

Brought into this form, it is observed that this invariant-based neighbour of the Ogden-model recovers the incompressible model proposed by Swanson (cf. eqn 13 in [87]) or, equivalently, the generalization of the *two-term* model of Lopez-Pamies (cf. eqn 23 in [88]). The principal stresses calculated from (5.15) are given by

5.17 $$\begin{array}{rl} P_{k}=\frac{nk_{B}\Theta }{3} & [\sum_{r=1}^{R^{+}}N^{(1-a_{r})/2}m_{r}\beta \lambda_{k}{\left(\frac{I_{1}}{3}\right)}^{\beta (a_{r}+1)/2-1}\\ & \;\;+\sum_{j=1}^{R^{-}}{\bar{m}}_{j}^{\prime }{\left(\frac{I_{2}}{3}\right)}^{{\bar{a}}_{j}/2-1}\lambda_{k}(I_{1}-\lambda_{k}^{2})]-p\lambda_{k}^{-1},\;k=1,2,3. \end{array}$$

Another eight-chain model is obtained if the chain stretch is defined through the non-affine mapping (3.2), which yields for each of the eight chains

5.18 $$\lambda_{r}={\left(\frac{I_{\beta }}{3}\right)}^{1/2}\;\mathrm{and}\;\nu_{r}={\left(\frac{I_{2}}{3}\right)}^{1/2},$$

where the notation

5.19 $$I_{\beta }={\text{U}}^{2\beta }:\text{I}={\text{C}}^{\beta }:\text{I}=\lambda_{1}^{2\beta }+\lambda_{2}^{2\beta }+\lambda_{3}^{2\beta }$$

has been introduced. In this case, the free energy reads

5.20 Ψ=nkBΘ[∑r=1R+MrAr[(Iβ3)Ar/2−1]+∑j=1R−m¯j′a¯j[(I23)a¯j/2−1]]

with corresponding principal stretches

5.21 $$\begin{array}{rl} P_{k}=\frac{nk_{B}\Theta }{3} & [\sum_{r=1}^{R^{+}}N^{(1-a_{r})/2}m_{r}\beta \lambda_{k}^{2\beta -1}{\left(\frac{I_{\beta }}{3}\right)}^{(a_{r}+1)/2-1}\\ & \;\;+\sum_{j=1}^{R^{-}}{\bar{m}}_{j}^{\prime }{\left(\frac{I_{2}}{3}\right)}^{{\bar{a}}_{j}/2-1}\lambda_{k}(I_{1}-\lambda_{k}^{2})]-p\lambda_{k}^{-1}, \end{array}$$

where k runs from 1 to 3. Although the first sum in equation (5.20) shares some features of its mathematical structure with a model proposed by Bechir [89] (see also [20]), it cannot be brought in complete agreement with the latter unless one enforces that β and $A_{r}/2$ are positive integers, most probably at the cost of either reduced capability to match experimental data or the need for more terms in the series.

### (c) Variation 3: non-affine chain deformation

The choice of ‘corotational’ chains implicit to the eight-chain or three-chain models here considered, whose end-to-end vectors transform with the stretch tensors rather than the deformation gradient, breaks with the concept of affine deformations *per se*. The same holds for average stretch concepts [25,51,59], which generally circumvent the definition of vector transformations. The power law (3.3), however, leads to a further relaxation of the affinity concept, and allows deviations from the affine stretch even for line elements along the principal directions.

Clearly, the most straightforward variation of the power law as a non-affinity concept is given by the choice β=1. In this case, Ogden’s model in the form (3.19) becomes a non-Gaussian three-chain model [45], albeit with a special representation of the non-Gaussian chain free energy so that

5.22 Ψ=nkBΘ3∑k=13[∑i=1R+MiAi(λkAi−1)+∑j=1R−m¯j′a¯j(λk−a¯j−1)].

However, that β<1 is needed to obtain favourable agreement with experimental data [16] suggests that this model may be of little practical use. Alternatives to the power-law concept for relaxing the affine stretch have been proposed, e.g. by Kroon [42] and Tkachuk & Linder [52]. Both approaches employ stationary principles to compute the non-affine stretch, and therefore do not lead to closed-form expressions of the free energy in terms of measures of deformation. A closed form of the free energy was obtained [90] by combining the idea of a maximal advance path [52] in the network with the average stretch concept [51]. The combination results in a non-affine stretch that scales with the functionality φ≥3 of the cross-links, i.e. the coordination number of the network, and provides the relation [90]

5.23 $$\lambda_{r}\mapsto \sqrt{\text{C}:{\text{H}}_{M}}.$$

The second-order tensor $H_{M}$ therein is defined as

5.24 $${\text{H}}_{M}=(1-3\kappa )M⊗M+\kappa \text{I},\;\kappa =2\frac{\varphi -1}{\varphi (\varphi +1)},$$

so that for any direction M the square stretch follows from a rule of mixture between the affine stretch square and the average stretch square $I_{1}/3$. In [90], this concept was applied to the full network, but in order to keep the changes with respect to the original model (3.19) as small as possible, the chain stretch is here evaluated for the three principal directions $N_{k}$, k=1,2,3. Replacing the chain stretch $\lambda_{k}^{\beta }$ in (3.19) with (5.23), the corresponding free energy reads

5.25 Ψ=nkBΘ3∑k=13[∑i=1R+MiAi[((1−3κ)λk2+κI1)Ai/2−1]+∑j=1R−m¯j′a¯j(λk−a¯j−1)],

while the constraint part was left unchanged. The nominal stresses along the principal directions read

5.26 $$\begin{array}{rl} P_{k} & \;=\frac{nk_{B}\Theta }{3}\left[\sum_{r=1}^{R^{+}}N^{(1-a_{r})/2}m_{r}{\left((1-3\kappa )\lambda_{k}^{2}+\kappa I_{1}\right)}^{(a_{r}-1)/2}\lambda_{k}(1-2\kappa )-\sum_{j=1}^{R^{-}}{\bar{m}}_{j}^{\prime }\lambda_{k}^{-{\bar{a}}_{j}-1}\right]-p\lambda_{k}^{-1}, \end{array}$$

where k again runs from 1 to 3.

### (d) Variation 4: topological constraints

The last variation concerns the representation of the free-energy contribution due to topological constraints, which in [16] was associated with those terms in Ogden’s model that have negative powers $\alpha_{r}$ and coefficients $\mu_{r}$, as summarized in §3c. In fact, alternative representations have already resulted naturally from a variation of the representative chains (§5b), albeit along with corresponding modifications of the free energy due to chain extension. Therefore, we here consider the constitutive model that is obtained when only those terms with negative powers and coefficients are varied and replaced by the constraint contribution proposed by Kroon [42], later also adopted in [59],

5.27 $$\Psi_{\nu }=nk_{B}\Theta c_{\mathrm{kr}}\left(\sqrt{\frac{I_{2}}{3}}-1\right),$$

in terms of the second principal invariant and a positive constant $c_{\mathrm{kr}}$. The thus modified Ogden strain-energy density expressed in the molecular statistical form reads

5.28 Ψ=nkBΘ[13∑k=13∑r=1R+MrArλkβAr+ckr(I23−1)].

The principal stresses are calculated as

5.29 $$P_{k}=\frac{nk_{B}\Theta }{3}\left[\sum_{r=1}^{R^{+}}N^{(1-a_{r})/2}m_{r}\beta \lambda_{k}^{\beta (a_{r}+1)-1}+c_{\mathrm{kr}}{\left(\frac{I_{2}}{3}\right)}^{-1/2}\lambda_{k}(I_{1}-\lambda_{k}^{2})\right]-p\lambda_{k}^{-1},$$

and k takes values from 1 to 3.

## 6. Comparison with experimental data

The goal of the present work is to highlight relations between Ogden’s and other models, both existing ones and new expressions that result from simple variations of the ingredients that provide the original strain-energy density function. A detailed discussion of these models with regard to their capacity for fitting sets of experimental data is beyond the scope of this paper. Nevertheless, in order to sketch the general characteristics predicted by the nine relatives of Ogden’s model obtained through a single variation, we calibrated their material parameters against a reproduction of Treloar’s data on vulcanized rubber [18] for illustration.

### (a) Parameter identification

Fitting the stress response of Ogden’s model [1] in homogeneous load cases to corresponding experimental data can lead to non-unique sets of the 2R parameters and is affected by the selection of the data used for calibration [46,91]. It has been shown that the molecular statistical reinterpretation of Ogden’s model [16] which comes with $3+2R^{-}$ parameters typically reduces the number of unknown parameters that need to be determined by comparison with experiments. For a given temperature, these parameters reduce to the chain density n, the number of links N, the non-affinity parameter β, and the constants $w_{\nu }\;{\bar{m}}_{j}$ and ${\bar{a}}_{j}$ which specify the tube geometry and deformation [16]. Given this clear interpretation of these parameters, they are either strictly bounded or at least restricted by physical reasonableness. This generally beneficial property carries over to the variations of the statistically motivated Ogden model studied herein, which largely share these parameters.

To determine these parameters, a custom Matlab (The Mathworks, Inc., v. R2018a) script was used to compute the principal stresses predicted by the different models under homogeneous states of deformation. We note that the discrete full-network model (5.12) was represented by M=88 directions on the unit sphere defining a spherical 12-design according to [92,93], whereas all other models are fully specified in §5, except for the number $R^{-}$ of terms in the constraint contribution. The latter was set to $R^{-}=1$ since this corresponds to a single term with negative power in Ogden’s model, which is typically needed for good agreement with experimental data [16]. We recall that the parameters $m_{r}$ and $a_{r}$ (and thus $M_{r}$ and $A_{r}$) are predefined and were set according to the $R^{+}=2$ approximations of the inverse Langevin function specified in [16]: $m_{1}=4.537$, $a_{1}=1.295$, $m_{2}=18.50$ and $a_{2}=10.98$.

Uniaxial tension (UA), equibiaxial tension (EB) and pure shear (PS) with prescribed tensile stretch $\lambda_{1}$ along a fixed direction $e_{1}$ were considered, where the deformation gradient $F=F_{ij}\;e_{i}⊗e_{j}$ was defined through the orthonormal basis $\left\{e_{1},e_{2},e_{3}\right\}$ and Cartesian components

6.1 $$\begin{array}{r} {}[F_{ij}^{\mathrm{UA}}]=\mathrm{diag}\left(\lambda_{1},\frac{1}{\sqrt{\lambda_{1}}},\frac{1}{\sqrt{\lambda_{1}}}\right),\;[F_{ij}^{\mathrm{EB}}]=\mathrm{diag}\left(\lambda_{1},\lambda_{1},\frac{1}{\lambda_{1}^{2}}\right)\;\mathrm{and}\;\;[F_{ij}^{\mathrm{PS}}]=\mathrm{diag}\left(\lambda_{1},1,\frac{1}{\lambda_{1}}\right), \end{array}$$

respectively. For all three states, the surface with normal $e_{3}$ was considered free of traction, so that $P_{3}=0$ allowed elimination of the arbitrary hydrostatic pressure p from the expression for the tensile nominal stress $P_{1}$. Considering the $N_{\mathrm{dat}}^{a}$ data pairs $\left(\lambda_{1,i}^{a},{\tilde{P}}_{1,i}^{a}\right)$ for each test mode (a=UA, PS, EB) in [18] and the corresponding stress $P_{1}^{a}(\lambda_{1,i}^{a})$ predicted by a model, the objective function

6.2 $$\begin{array}{r} {\bar{\varepsilon }}^{2}=\sum_{a=\mathrm{UA},\mathrm{EB},\mathrm{PS}}\frac{1}{N_{\mathrm{dat}}^{a}}\sum_{i=1}^{N_{\mathrm{dat}}^{a}}{\left[\frac{P_{1}^{a}(\lambda_{1,i})-{\tilde{P}}_{1,i}^{a}}{{\tilde{P}}_{1,i}^{a}}\right]}^{2} \end{array}$$

was minimized by means of the Matlab function fmincon. Although a detailed analysis of the parameters’ uniqueness and sensitivity is beyond the scope of the present work, the fitting routines were initiated several times with different starting parameters according to the following scheme. When present in a model, the values of the parameters in the set $P_{0}=\{nk_{B}\Theta ,N,\beta ,{\bar{m}}_{1}^{\prime },{\bar{a}}_{1}\}$ given in [16] were used as initial guesses at first. Then the routine was repeated another S=30 times upon applying a random 20% variation of the initial set of parameters and restarting the optimization with the new initial guess $P_{s}$, s=1,2,…,30. The optimized set of parameters was then identified from the procedure that led to the smallest value of ${\bar{\varepsilon }}^{2}$. For the sake of comparability, a coefficient of determination $R^{2}$ applicable to nonlinear models [94] was calculated for each of the nine variations and every mode a={UA,EB,PS}. Even if approximate, it serves as a scalar indicator of the goodness of fit and allows coarse comparison of the models.

### (b) Results

**Figure 5.**
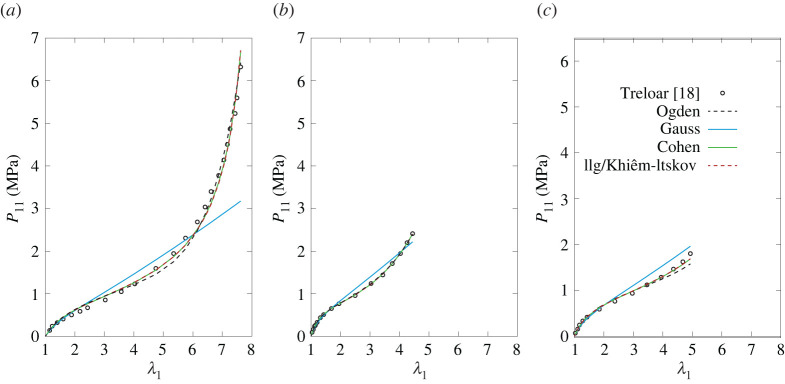
Nominal stress in UA (*a*), EB (*b*) and PS (*c*) homogeneous deformation states for the models obtained by varying the chain model (variation 1). The statistical Ogden’s model with $R^{+}=2$ and $R^{-}=1$ is plotted for comparison (with parameters from [16]). (Online version in colour.)

**Figure 6.**
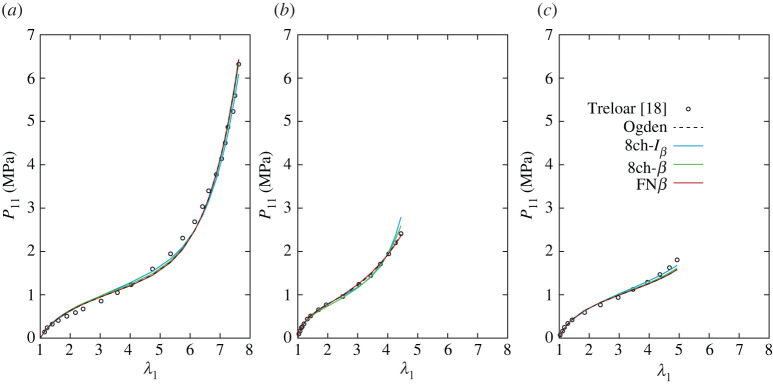
Nominal stress in UA (*a*), EB (*b*) and PS (*c*) homogeneous deformation states for the models obtained by varying the representative chains (variation 2). The statistical Ogden’s model with $R^{+}=2$ and $R^{-}=1$ is plotted for comparison (with parameters from [16]). (Online version in colour.)

**Figure 7.**
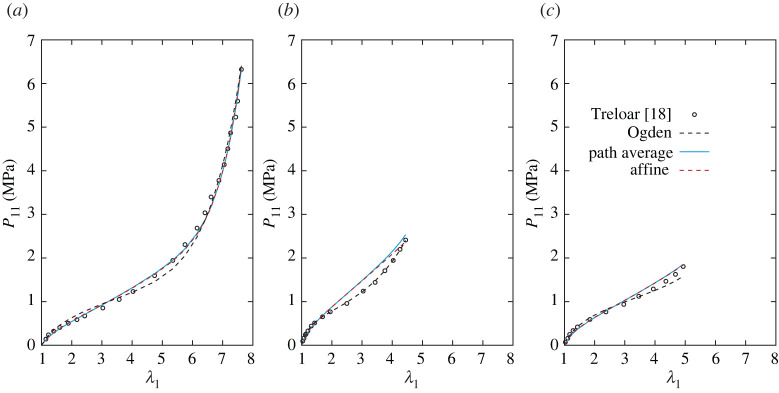
Nominal stress in UA (*a*), EB (*b*) and PS (*c*) homogeneous deformation states for the models obtained by considering different approaches to accounting for non-affinity (variation 3). The statistical Ogden’s model with $R^{+}=2$ and $R^{-}=1$ is plotted for comparison (with parameters from [16]). (Online version in colour.)

**Figure 8.**
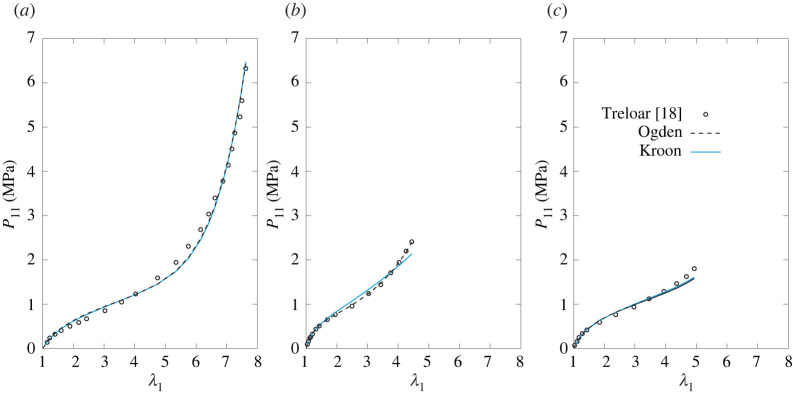
Nominal stress in UA (*a*), EB (*b*) and PS (*c*) homogeneous deformation states for the model obtained by varying the contribution of the topological constraint (variation 4). The statistical Ogden’s model with $R^{+}=2$ and $R^{-}=1$ is plotted for comparison (with parameters from [16]). (Online version in colour.)

Tables 1–4 report the values of the parameters estimated through the optimization routine, and, for comparison, the parameters of the unvaried statistical representation of the Ogden model [16] are reported in the first row of each table. The corresponding plots are provided in figures 5–8. The exemplary application of the models to the classical benchmark dataset of Treloar can clearly not testify to their capacity to fit the elastic response of the broad range of rubber-like materials. However, it provides an indication of how well they capture the principal characteristics of these materials on the one hand, while highlighting differences from Ogden’s model on the other.

**Table 1.** Fiitted parameters for the models obtained by varying the chain model (variation 1).

model	equation	$nk_{B}\Theta \;[\mathrm{MPa}]$	N	β	${\bar{m}}_{1}^{\prime }$	${\bar{a}}_{1}$
Ogden	(3.19)	0.7495	18.69	0.6992	0.01025	2.518
Gauss	(5.4)	0.2305	—	1.1154	1.7932	1
Cohen	(5.6)	0.70308	22.5883	0.73053	0.011241	2.4712
Ilg/Khiêm–Itskov	(5.9)	0.68877	23.1587	0.73859	0.011098	2.4808

A closer analysis reveals that, irrespective of the number and disposition of the representative chains, consideration of a non-affine relation of the chain stretch in terms of a β-power law, adopted for the non-Gaussian approximations (5.6), (5.9), (5.12), (5.15) and (5.20), generally improves the simultaneous fitting of the three homogeneous deformation states. The use of the affine stretch (5.22), even in an average sense, i.e. $\sqrt{I_{1}/3}$ as in (4.2) or their combination in the path average as in (5.25), indicates somewhat less accurate fitting capacities.

In this regard, one may also note the value of N that characterizes the length Nℓ of the freely jointed chain. The value obtained by parameter identification is considerably higher for the affine model (5.22) (N≈74) than in the non-affine cases, and it seems obvious that this large value is necessary to postpone the strong increase in stiffness when one of the principal stretches approaches the limit $\lambda_{k}\to \sqrt{N}$.

Finally, it is interesting to compare the parameters ${\bar{m}}_{1}^{\prime }$ and $c_{\mathrm{kr}}$ that weight the contribution of the tube constraint and that vary over several orders of magnitude, from essentially zero for the $I_{\beta }$ model (table 2) to a substantial weight in the path average and affine models (table 3), where the fitted power ${\bar{a}}_{1}$ takes the lowest limit allowed. The $I_{\beta }$ model, on the other hand, thereby effectively becomes a three-parameter model that excellently fits the experimental data of Treloar.

**Table 2.** Fitted parameters for the models obtained by varying the representative chains (variation 2).

model	equation	$nk_{B}\Theta$ (MPa)	N	β	${\bar{m}}_{1}^{\prime }$	${\bar{a}}_{1}$
Ogden	(3.19)	0.7495	18.69	0.6992	0.01025	2.518
FNβ	(5.12)	0.58029	16.0777	0.72483	0.057743	2.0182
8ch-β	(5.15)	0.49899	10.0428	0.73799	0.049652	2
8ch-$I_{\beta }$	(5.20)	0.58202	9.808	0.78778	$2.2204\times 10^{-16}$	2.4053

**Table 3.** Fitted parameters for the models obtained by considering different approaches to accounting for non-affinity (variation 3). Note that the functionality φ was set to 4 in the path average model.

model	equation	$nk_{B}\Theta$ (MPa)	N	β	${\bar{m}}_{1}^{\prime }$	${\bar{a}}_{1}$
Ogden	(3.19)	0.7495	18.69	0.6992	0.01025	2.518
affine	(5.22)	0.25275	74.0452	—	2.6156	1
path average	(5.25)	0.64547	30.0567	—	0.88761	1

**Table 4.** Fitted parameters for the model obtained by varying the contribution of the topological constraint (variation 4).

model	equation	$nk_{B}\Theta$ (MPa)	N	β	${\bar{m}}_{1}^{\prime }$	${\bar{a}}_{1}$	$c_{\mathrm{kr}}$
Ogden	(3.19)	0.7495	18.69	0.6992	0.01025	2.518	—
Kroon	(5.28)	0.59893	24.3985	0.75854	—	—	0.11637

With the exception of the Gaussian chain variation (5.4), which is inadequate for describing the large strain regime as expected, the overall agreement of the variations with the triplet of experimental data is generally high. Table 5 summarizes the coefficients of determination $R^{2}$ of the fitted models for each test mode and documents rather small differences between the remaining eight models.

**Table 5.** $R^{2}$ values for each model and each deformation state, calculated according to [94].

	Ogden	Gauss	Cohen	Ilg	FNβ	8ch-β	8ch-$I_{\beta }$	affine	path avg.	Kroon
$R_{\mathrm{UA}}^{2}$	0.9940	0.6668	0.9939	0.9930	0.9937	0.9953	0.9960	0.9970	0.9976	0.9933
$R_{\mathrm{EB}}^{2}$	0.9997	0.9838	0.9998	0.9998	0.9991	0.9947	0.9815	0.9689	0.9595	0.9847
$R_{\mathrm{PS}}^{2}$	0.9801	0.9466	0.9950	0.9950	0.9804	0.9859	0.9928	0.9870	0.9858	0.9856

## 7. Concluding remarks

In the present contribution, we have used a recently proposed statistical interpretation of Ogden’s model to identify closely related models by a single variation of its basic ingredients. The schematic of the way this and other models can be derived from statistical mechanical concepts and the idea of representing the polymer network through a number of representative chains show that these models share the same essential steps in their development. Although they can thus be categorized into the same large family of models, there are very few known close relatives that differ from the statistical version of Ogden’s model only in a single step. In fact, we have discovered only one such model, *viz*. the strain-energy density function (5.15), which is a special case of the hyperelastic constitutive equations proposed in [87,88].

A main reason for this special position of Ogden’s model is the relaxation of the affine assumption through a power law. In general, this operation turns out to be beneficial since nearly all variations containing this step have shown very sound agreement with the experimental dataset.

In summary, Ogden’s model—even if it can be well embedded in the family of hyperelastic models with molecular statistical meaning—remains unique in the combination of its ingredients.

## Data Availability

All the data used in the present work are provided as data plots in the figures.
